# Evaluating User Experience and Satisfaction in a Concussion Rehabilitation App: Usability Study

**DOI:** 10.2196/67275

**Published:** 2025-04-11

**Authors:** Michael G Hutchison, Alex P Di Battista, Kyla L Pyndiura

**Affiliations:** 1 Faculty of Kinesiology and Physical Education, University of Toronto, 100 Devonshire Pl, Toronto, ON, M5S 2C9, Canada, 1 416 946 5630; 2 David L. MacIntosh Sport Medicine Clinic, Faculty of Kinesiology & Physical Education, University of Toronto, Toronto, ON, Canada; 3 Centre for Sport-Related Concussion Research, Innovation, and Knowledge, University of Toronto, Toronto, ON, Canada; 4 Defence Research and Development Canada, Toronto Research Centre, Toronto, ON, Canada

**Keywords:** mild traumatic brain injury, recovery, mHealth, app, digital health, smartphone, eHealth, digital, technology, usability, concussion rehabilitation, brain injury, rehabilitation protocols, evidence-based exercise, single-arm pilot study, home-based rehabilitation, user-friendly, questionnaire, telehealth, telemedicine

## Abstract

**Background:**

Evidence-based guidelines support the use of structured exercise to facilitate concussion recovery. Despite the growing number of mobile health (mHealth) apps aimed at managing concussions, few focus on delivering exercise rehabilitation protocols. Therefore, a mobile app was developed to provide personalized rehabilitation programs based on evidence-based exercise principles designed to cater to individuals recovering from concussions.

**Objective:**

This study aimed to evaluate the usability and user experience of a mobile app designed to deliver an evidence-based rehabilitation program to individuals recovering from concussions.

**Methods:**

A two-week prospective single-arm pilot study was conducted among adults with a physician-diagnosed concussion. Participants engaged in home-based rehabilitation exercises through a mobile app. Usability was assessed using a combination of the mHealth App Usability Questionnaire (MAUQ) and five custom questions evaluating confidence in recommendations, exercise flow, clarity of voice commands, and usability of the exercise report feature. Following the two-week period, participants rated each question on a 7-point Likert scale ranging from “strongly agree” to “strongly disagree”.

**Results:**

Twenty-six participants consented and were enrolled in the study, with 23 participants (82%) completing all study components at the end of two weeks. The majority of participants were women, aged 26-38 years, and on average, approximately at three months postconcussion. Responses to both the MAUQ and custom questions were overwhelmingly positive. Overall, seven MAUQ questions received 100% positive responses, with no single question scoring below 83% positive responses. In the “ease of use and satisfaction” category, 100% of users responded positively to questions on ease of learning, usability, interface likeness, and comfort in social settings, while 83%-96% (19-22) of users responded positively to the remaining four questions. In the “system information arrangement” category, 100% (N=23) of users rated screen navigation, function usability, and health care service acceptability positively, with 87%-96% of users approving action acknowledgment, error recovery, and expected functions. Under the “usefulness” category, 96% (n=22) of users found the app beneficial for health and well-being, and 91% (n=21) users felt it effectively managed their health. For the five custom questions, 100% (N=23) users responded positively to voice command clarity, exercise awareness, ease of following exercises, and report understandability, with a single unfavorable response noted for confidence in app recommendations.

**Conclusions:**

The findings of this study indicate that the mobile app is a user-friendly platform for delivering evidence-based exercise rehabilitation to individuals recovering from concussions. Positive user feedback, particularly in the areas of recommendation confidence, ease of exercise flow, and clarity of voice commands, highlights the app’s potential to support concussion recovery. Future iterations of the app will aim to improve time efficiency and streamline error recovery processes to further enhance the user experience.

## Introduction

Concussion is a common form of traumatic brain injury (TBI) that can occur in a variety of settings such as sports, motor vehicle accidents, workplaces, and daily life. Symptoms range from physical and cognitive disruptions to emotional and sleep disturbances [1], underscoring the highly individualized nature of the injury. Following an initial 24‐ to 48-hour period of cognitive and physical rest, research has demonstrated the benefits of aerobic exercise in enhancing concussion recovery. This approach not only alleviates symptom severity but can also expedite the recovery process [2-4].

The recommendation of aerobic exercise as an intervention for concussion often involves establishing a personalized heart rate (HR) threshold set by trained professionals [5,6]. More recently, the determination of exercise intensity has evolved to include the use of age-predicted maximum HR formulas [3], offering a more accessible and individualized approach to concussion management. However, to enhance the impact of these advancements, it is crucial to integrate exercise protocols into clinical practices beyond sports and exercise medicine. This can ensure that a wider patient population benefits from the most effective, evidence-based strategies for concussion management, thus achieving a more comprehensive and equitable standard of care.

Mobile health (mHealth) has emerged as a widely accessible medium for individuals to obtain medical care. This technology is particularly beneficial for individuals in remote areas or those who face challenges in accessing health care facilities. Consequently, there has been a surge in the development of health care–focused mobile apps in recent years, demonstrating significant benefits. Notably, mHealth interventions in mental health have shown promise in reducing symptoms of depression and anxiety across diverse population groups [7,8]. Additionally, mHealth apps have been effectively used in various health care domains, including cardiovascular disease prevention and diabetes management, showcasing their versatility and impact on modern health care [9-12].

The application of mHealth is increasingly recognized for its potential benefits in managing TBI. Various mobile apps aimed at providing education about TBI, symptom tracking, daily reminders for patients with TBI, and aiding in the screening or assessment of TBI are now available [13-15]. Despite these advancements, a notable gap exists in apps specifically tailored for the management of TBIs [13]. Moreover, many of these apps have yet to achieve widespread user adoption; a significant concern is that many available apps lack evidence-based interventions, making it difficult for health care providers to recommend them and for patients to use them with confidence [13]. In concussion care, the primary focus of mobile apps has been on symptom reporting, monitoring activity levels, and concussion recognition targeted at parents, coaches, athletic trainers, and athletes [16-18]. However, current apps do not focus on interventions involving exercise or physical activity, indicating an area for further development in mHealth solutions for TBI and concussion management.

Hutchison et al [19] conducted a study evaluating the feasibility of a mobile app designed to deliver resistance exercises using minimal equipment for individuals recovering from concussions. The app provides an aerobic stimulus by guiding users to achieve a percentage of their age-predicted maximal HR through tailored exercise routines. This innovative tool includes instructional videos to assist users in their rehabilitation process. Initial results demonstrated that most participants were able to reach the target HR zones by following the app-based exercise videos [19]. Building on these preliminary findings, which focused on the app’s ability to elicit the desired physiological responses [19], the present study aims to expand on this research by assessing the app’s overall usability. Specifically, we sought to identify potential areas for improvement, gauge user satisfaction, and evaluate the app’s effectiveness in supporting concussion recovery.

## Methods

### Study Design

The usability study consisted of a two-week, community-oriented, prospective single-arm observational pilot study involving adults aged 18 years and older with a physician-diagnosed concussion. Participants were engaged in an active, home-based rehabilitation program accessed through the mobile app. Individuals were recruited through word-of-mouth, flyers, and social media platforms (eg, X, formerly known as Twitter, Facebook) and were provided detailed information regarding study requirements and procedures. Participant enrollment was conducted remotely via Zoom, and individuals were screened by a research assistant to confirm inclusion criteria consisting of a concussion diagnosis by a physician, aged at least 18 years, and access to an iPhone. Furthermore, individuals with coexisting conditions such as musculoskeletal or soft-tissue injuries, vestibular disorders, or neurological conditions that could impede physical activity, were excluded from the study. Twenty-six individuals consented and enrolled in the study. Upon enrollment, participants were mailed an Apple Watch Series 6 to use with their iPhones and access to the Rhea Health Inc. mobile app. Ultimately, 23 participants completed the usability questionnaire after using the mobile app for two weeks.

### Ethical Considerations

This study received ethical approval from the Health Sciences Research Ethics Board at the University of Toronto (protocol #39143). To ensure comprehensive understanding and informed decision-making, prospective participants were given an information sheet by a member of the research team. This sheet detailed the study’s eligibility criteria, purpose, methodologies, and any potential risks involved. Before participants gave their consent, their eligibility was confirmed via email. A Zoom videoconference was then conducted to clarify any queries regarding the study and reinforce the voluntary nature of participation and the freedom to withdraw at any point without adverse consequences. Informed consent was secured through an electronic process in which participants reviewed and signed a digital document that outlined the study’s purpose, procedures, and potential risks. This consent form was then submitted using the REDCap (Research Electronic Data Capture) platform, hosted by the University of Toronto.

Data collected via the mobile app and Apple Watch were securely stored on the Amazon Web Services’ DynamoDB database, located in Montreal, Quebec, Canada. This storage solution employed Amazon Web Services’ proprietary encryption and restricted access system to ensure data security. Although participants were required to use their emails and passwords for accessing the mobile app and syncing data from their Apple Watch to Apple Health, personally identifiable information was stored in a separate database table and was not required for data analysis.

Upon the study’s completion, participants were compensated CAD $50 (equivalent to approximately US $37.16) for their participation and were entered into a lottery for a chance to win an Apple Watch Series 6. This gesture not only acknowledged their contribution but also encouraged engagement and retention in the study.

### The Mobile App

**Figure 1.**
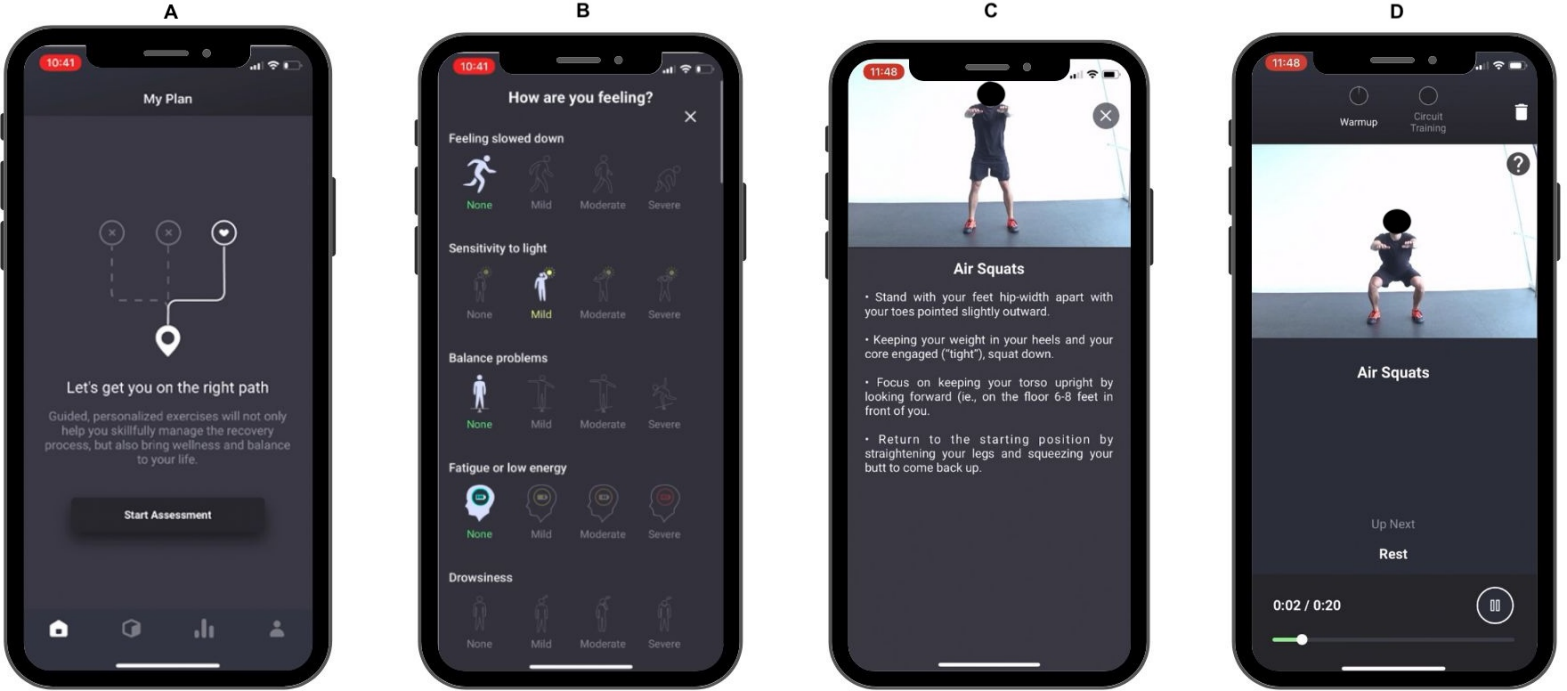
Screenshots of the Rhea mobile app from left to right display (A) the home page of a participant’s plan, (B) a symptom checklist during onboarding that assists with exercise prescription, (C) an exercise preview screen providing description and cues for correct form, and (D) an exercise session screen with a timer and details of the next exercise for the user.

The rehabilitation protocol in this study was delivered through the Rhea mobile app (Rhea Health Inc.) (Figure 1), a rehabilitation platform that devises personalized programs based on an individual’s symptoms and time elapsed since injury. The app integrates symptom data, including 22 items from the Sports Concussion Assessment Tool (SCAT-5) [20], along with seven additional questions related to vision and balance. Participants assessed their symptom severity using a 4-point Likert Scale, ranging from 0 (none) to 3 (severe). Following a staged approach endorsed by consensus statements on concussion management, the Rhea rehabilitation protocol employed a six-stage model that progressively increased in intensity and complexity. These stages extend from Initial Rest (Stage 1) to Return (Stage 6). Participants commenced their rehabilitation at Stage 2, completing three sessions at this level before progressing. One of the Rhea app’s unique features is its dynamic adjustment of the rehabilitation plan that is based on participant feedback, which categorizes symptomology as “same,” “better,” or “worse.” This feedback-driven mechanism ensures that the participants either stay at their current stage, advance to a more challenging stage, or revert to a less intensive stage, as required. The primary objective of this adaptive approach is to fine-tune the intensity and complexity of exercises to avoid exacerbating symptoms, thus promoting a safe and effective journey through the recovery stages. In addition to the six core stages, Rhea’s program provided participants with exercises specifically tailored to neck, vision, and balance rehabilitation. These exercises are designed to address each participant’s unique symptom profile, further personalizing the recovery experience. This approach not only ensures adherence to the rehabilitation protocol but also maximizes the potential for effective recovery tailored to individual needs. To monitor adverse events, participants were instructed to inform a member of the research team of any adverse events (eg, injuries, medical conditions) that were experienced while using the mobile app.

### Usability Questionnaire

The mHealth App Usability Questionnaire (MAUQ) [21] contains 21 questions rated on a 7-point Likert scale, ranging from strongly agree to strongly disagree, under three categories: ease of use and satisfaction, system information arrangement, and usefulness. Studies have identified the MAUQ to be a reliable and valid tool for assessing mHealth app usability [21]. In this study, we used 16 questions from the MAUQ and omitted the 5 questions from the usefulness category, as they assess aspects related to sharing information with health care providers and clinical utility. Due to the preliminary nature of the research and the use of a research-grade tool that lacked standards and approval for clinical use, we did not ask or expect participants to engage with health care professionals using the tool. Additionally, we included five custom questions to evaluate the app’s critical features related to the delivery of exercise rehabilitation, as the app was based on evidence-based exercise rehab principles. These custom questions were designed to assess user confidence in the app’s recommendations, clarity of exercise instructions, the flow of the exercise content, and the usability of the app’s voice commands. More specifically, given the central role of accurate exercise prescription in rehabilitation, it was important to gauge whether users trusted the app’s recommendations; therefore the question, *“*I had confidence in the recommendations I received from the app*”* was included. Since effective rehabilitation relies on the clarity and ease of exercise guidance, we included two questions targeting the flow and clarity of the exercise sessions: *“*The flow of the exercise sessions on the app is easy to follow*”* and *“*I knew exactly which exercises I had to do during an exercise session*.”* These questions allowed us to determine whether participants found the session progression intuitive and well-structured. We also added a question about voice commands: *“*The in-app voice commands are clear and understandable*.”* This was a critical feature for users who might benefit from auditory guidance, particularly those experiencing cognitive fatigue or visual sensitivity due to their concussion. Clear voice commands would support a hands-free interaction and reduce the cognitive load associated with reading instructions on a screen. Lastly, we evaluated the app’s reporting functions through the question, *“*The exercise report section is easy to follow and understand*.”* A clear and accessible exercise report is vital for tracking progress and ensuring participants understand their rehabilitation. These custom questions were specifically tailored to capture the nuances of delivering exercise rehabilitation through a digital platform, ensuring that the app was not only usable but also effective in guiding users through their recovery process.

### Data Analysis

The purpose of the current study was to summarize and report participant feedback following a two-week period of using the mobile app. Therefore, the Likert-scale responses from the MAUQ and additional customized app usability questions were summarized and reported as raw counts and percentages. For additional continuous variables, the median (IQR) were calculated.

## Results

### Participant Characteristics

Study participant characteristics can be seen in Table 1. Briefly, the sample comprised predominantly of female participants (n=18, 78%), aged 26‐38 years. On average, the participants were nearly three months post-concussion, with 48% (n=11) sustaining injury during sports or physical activity.

**Table 1.** Demographic and clinical characteristics of participants, including age, sex, mechanism of injury, and time since concussion.

Characteristics	Participants (N=23)
Sex, n (%)	
Female	18 (78)
Male	5 (22)
Age, median (IQR)	28.4 (25.1-37.3)
Days from injury, median (IQR)	82 (25-461)
Mechanism of injury, n (%)	
Sports/PA^a^	11 (48)
MVC^b^	1 (4.3)
Work	1 (4.3)
Fall	2 (8.7)
Other	8 (35)
History of concussion, n (%)	13 (59)
High performance athlete, n (%)	5 (22)
Exercise program, n (%)	8 (35)
Physical activity (times/week)	
0	3 (13)
1	2 (8.7)
2	4 (17)
3	3 (13)
4	2 (8.7)
5	4 (17)
6	3 (13)
≥7	2 (8.7)

a PA: physical activity.

b MVC: Motor vehicle collision.

### Concussion Characteristics

Participants entered the study with an average of 14 symptoms and a symptom severity score of 29. On average, they reported feeling at approximately 70% of their usual health, and 43% (n=10) of the participants stated that they were getting better following their injury at first, but later experienced symptom worsening. Please see Table 2 for the complete symptom profiles of the participants.

**Table 2.** Symptom profile at onboarding, including symptom severity, perceived normalcy, and symptom progression trends.

Characteristics	Participants (N=23)
Symptom number, median (IQR)	14 (11-18)
Symptom severity, median (IQR)	29 (19-44)
Perceived percent normalcy, median (IQR)	70 (50-75)
Trends in symptoms since injury, n (%)	
No change	1 (4.3)
Steadily better	8 (35)
Steadily worse	1 (4.3)
Worse, then better	3 (13)
Better, then worse	10 (43)

### MAUQ Findings

**Figure 2.**
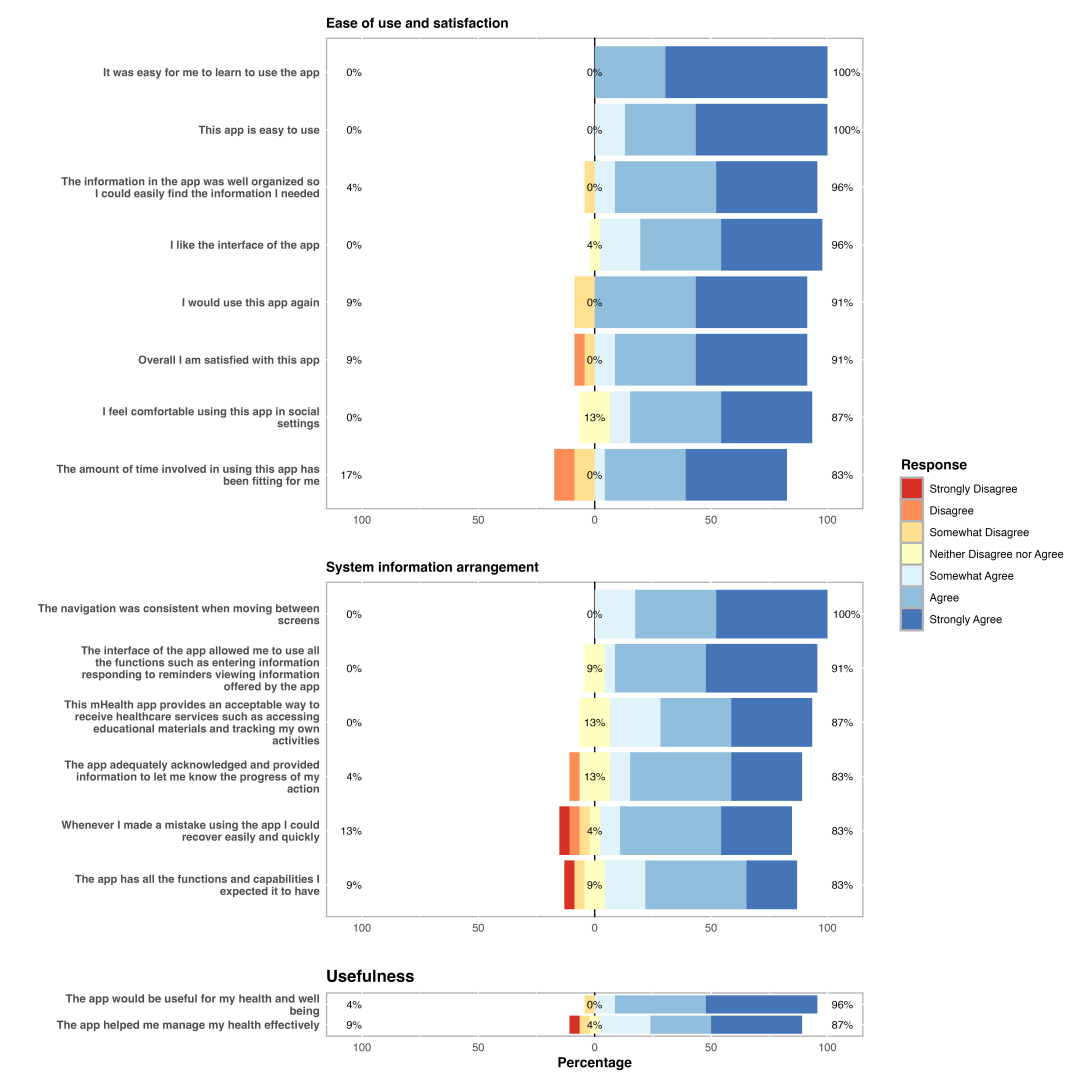
Responses from participants to each of the 16 mHealth app usability questionnaire (MAUQ). Participants ranked each question using a 7-point Likert scale ranging from strongly agree (dark blue) to strongly disagree (red). Each horizontal bar indicates the breakdown of each question’s responses as percentages.

Participants’ responses to the MAUQ can be seen in Figure 2. Across the 16 MAUQ questions, “agree” or “strongly agree” were the most common responses. Under the “ease of use and satisfaction” category, all 23 participants selected “agree” or “strongly agree” for the question “easy to learn.” Twenty participants (87%) selected “agree” or “strongly agree” for both “easy to use” and “well organized” questions. The question with the least favorable response in this category was “appropriate time involved,” where 4 participants (17%) selected “disagree” or “somewhat disagree.”

Under the category “system information arrangement,” 20 (87%) participants selected “agree” or “strongly agree” for the question “all functions usable,” and 19 (87%) for the question “screen navigation consistent.” The most unfavorably responded question for this category was “ease of recovery following mistake,” where 3 participants (13%) chose either “strongly disagree,” “somewhat disagree,” or “disagree.”

In the “usefulness” category, 20 (87%) participants responded with “agree” or “strongly agree” for “health and well-being usefulness,” and 21 participants (87%) reported similarly for “helped manage health.” Please see Multimedia Appendix 1 for a summary of participant responses to each question of the MAUQ.

### Additional Customized Questions

**Figure 3.**
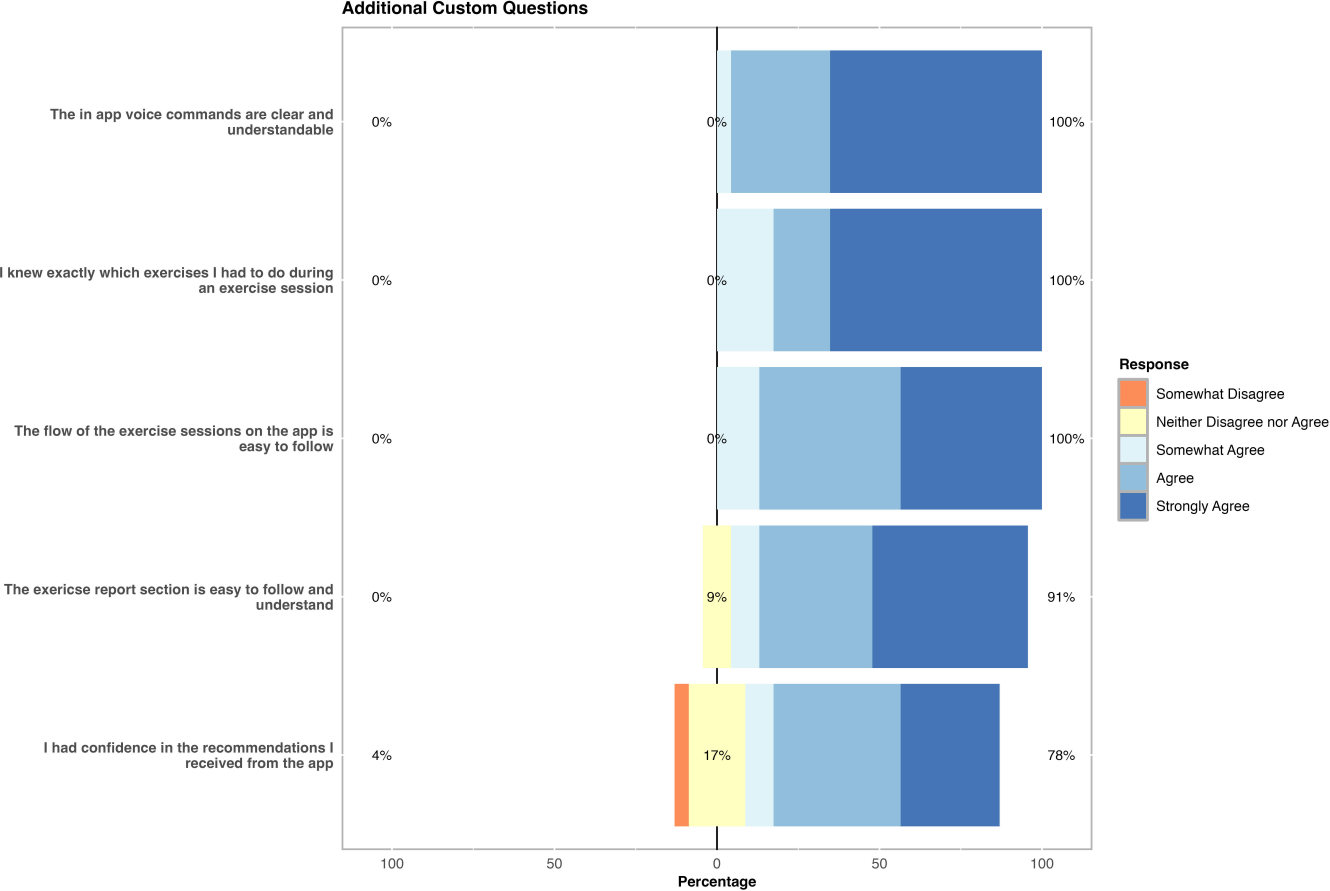
Participants’ responses to five additional custom questions. Participants ranked each question using a 7-point Likert scale ranging from strongly agree to strongly disagree; however, only 5 responses were chosen by participants, ranging from strongly agree (dark blue) to somewhat disagree (orange). Each horizontal bar indicates the breakdown of each question’s responses as percentages.

The additional questions specific to the app yielded similar responses to the MAUQ, with “agree” or “strongly agree” being the most common response for all five questions (Figure 3); only a single negative response was reported across all questions. The most favorable response was for “voice command clarity” where all but one participant (n=22, 95%) reported either “agree” or “strongly agree”; the other participant selected “somewhat agree.” Please see Multimedia Appendix 2 for a summary of the participant responses for the additional questions.

## Discussion

This study aimed to assess the usability of a mobile app that provides exercise rehabilitation for individuals with persistent concussion symptoms. Usability was assessed using a combination of the MAUQ and additional custom questions. Overall, the responses were strongly positive, indicating that participants found the app both useful and easy to navigate.

The app’s core features were well received by users, as indicated by the absence of disagreements for seven of the MAUQ questions. Responses to these questions were either neutral or positive among participants, which strongly supports the overall user satisfaction with the app. The most favorable responses were primarily in the “ease of use and satisfaction” category, followed closely by the “system information arrangement” category. This positive feedback is particularly significant for individuals recovering from concussions, as symptoms such as cognitive complaints (eg, difficulty concentrating) and sensitivity to screens and light are commonly reported [22-24]. Providing a mobile app that is easy to navigate and well organized is essential for enhancing adoption and encouraging consistent use throughout the recovery process.

The strongly positive feedback based on the custom questions provides evidence that the mobile app effectively meets the needs of individuals recovering from concussions. Participants reported high confidence in the app’s recommendations, which is particularly significant given that the app is grounded in evidence-based exercise rehabilitation principles. Confidence in the accuracy and safety of the recommendations is critical in concussion recovery, as missteps in physical activity intensity can lead to symptom exacerbation or worsening of neurocognitive performance [25]. The intuitive flow of exercise sessions and the clarity in guiding participants on which exercises to perform suggest that the app successfully translates complex rehabilitation protocols into an easy-to-follow format. Additionally, including clear voice commands helps reduce cognitive load, making the app accessible even to those with cognitive fatigue or visual sensitivity. Furthermore, the ease of understanding the exercise report section implies that users could track their progress effectively, promoting engagement and adherence to the rehabilitation protocol. These findings underscore the importance of mHealth solutions that provide evidence-based care and inspire user confidence, ultimately enhancing the recovery experience for concussion patients.

While participants generally found the app easy to use, our study revealed concerns regarding the time commitment and error recovery processes, both of which require further attention. Specifically, some participants found the app’s required time commitment to be excessive, and others experienced difficulty with the app’s response to user errors. To address these concerns, future developments of the app will aim to find an optimal balance between screen time and cognitive load. This balance is crucial for supporting individuals with concussions, who may be particularly sensitive to long periods of screen interaction and cognitive stress. Enhancements will include more efficient navigation and task completion processes, as well as integrated educational features that prompt users to take necessary breaks and provide guidance on managing screen time effectively. Moreover, improving how the app handles mistakes—making error recovery more intuitive and less stressful—is essential for fostering a supportive and rehabilitative environment. These adjustments will not only enhance the user experience but also play a pivotal role in the app’s functionality, ensuring it meets the unique needs of its users during their recovery.

There are several limitations to this study. First, the sample comprised predominantly female participants, which may limit the generalizability of the findings to male participants. Future studies should aim for more balanced demographics to validate the app’s usability across diverse populations. Additionally, our sample consisted of individuals with persistent symptoms, leaving the usability of the app in the acute and subacute phases of concussion recovery still unexplored. Larger-scale studies with a more diverse population are necessary to further confirm the app’s usability and effectiveness across different recovery stages. It is also important to note that we intentionally excluded five questions from the original MAUQ due to the early iteration of this tool. Specifically, we chose not to assess aspects related to sharing information with health care providers and clinical utility, as practitioners were not expected to engage with a research-grade tool at this stage. As the app evolves, future research should include these additional questions to better understand how the app complements health care practitioners and facilitates integration into clinical practice.

In conclusion, the Rhea app represents a promising mHealth solution for concussion rehabilitation. The overwhelmingly positive usability feedback supports the potential for broader adoption. However, continued refinement based on user feedback and further research with larger, more diverse populations is essential to ensure the app’s long-term success in facilitating concussion recovery.

## Supplementary material

10.2196/67275 Multimedia Appendix 1 Summary of participants’ responses to MAUQ questions.

10.2196/67275 Multimedia Appendix 2 Summary of participants’ responses to additional custom questions.

## Abbreviations

HR: heart rate
MAUQ: mHealth app usability questionnaire
mHealth: mobile health
SCAT-5: sports concussion assessment tool
TBI: traumatic brain injury
