# Supplementary material for: Evaluating User Experience and Satisfaction in a Concussion Rehabilitation App: Usability Study
Source: JMIR Form Res. 2025 Apr 11;9:e67275. doi: 10.2196/67275 (PMC12007725; doi:10.2196/67275)
Supplement: Multimedia Appendix 1 [file formative-v9-e67275-s001.docx]

*Multimedia Appendix 1. Summary of responses by participants with concussion to MAUQ questions following a 2-week use period of a personalized concussion rehabilitation mobile application.*

| **Characteristic** | **Strongly Disagree***^1^* | **Disagree***^1^* | **Somewhat Disagree***^1^* | **Neither Disagree nor Agree***^1^* | **Somewhat Agree***^1^* | **Agree***^1^* | **Strongly Agree***^1^* |
| --- | --- | --- | --- | --- | --- | --- | --- |
| **Ease of use and satisfaction (N = 23)** | | | | | | | |
| This app is easy to use | 0 (0%) | 0 (0%) | 0 (0%) | 0 (0%) | 3 (13%) | 7 (30%) | 13 (57%) |
| It was easy for me to learn to use the app | 0 (0%) | 0 (0%) | 0 (0%) | 0 (0%) | 0 (0%) | 7 (30%) | 16 (70%) |
| I like the interface of the app | 0 (0%) | 0 (0%) | 0 (0%) | 1 (4.3%) | 4 (17%) | 8 (35%) | 10 (43%) |
| The information in the app was well organized so I could easily find the information I needed | 0 (0%) | 0 (0%) | 1 (4.3%) | 0 (0%) | 2 (8.7%) | 10 (43%) | 10 (43%) |
| I feel comfortable using this app in social settings | 0 (0%) | 0 (0%) | 0 (0%) | 3 (13%) | 2 (8.7%) | 9 (39%) | 9 (39%) |
| The amount of time involved in using this app has been fitting for me | 0 (0%) | 2 (8.7%) | 2 (8.7%) | 0 (0%) | 1 (4.3%) | 8 (35%) | 10 (43%) |
| I would use this app again | 0 (0%) | 0 (0%) | 2 (8.7%) | 0 (0%) | 0 (0%) | 10 (43%) | 11 (48%) |
| Overall I am satisfied with this app | 0 (0%) | 1 (4.3%) | 1 (4.3%) | 0 (0%) | 2 (8.7%) | 8 (35%) | 11 (48%) |
| **System information arrangement (N = 23)** | | | | | | | |
| Whenever I made a mistake using the app I could recover easily and quickly | 1 (4.3%) | 1 (4.3%) | 1 (4.3%) | 1 (4.3%) | 2 (8.7%) | 10 (43%) | 7 (30%) |
| This mHealth app provides an acceptable way to receive healthcare services such as accessing educational materials and tracking my own activities | 0 (0%) | 0 (0%) | 0 (0%) | 3 (13%) | 5 (22%) | 7 (30%) | 8 (35%) |
| The app adequately acknowledged and provided information to let me know the progress of my action | 0 (0%) | 1 (4.3%) | 0 (0%) | 3 (13%) | 2 (8.7%) | 10 (43%) | 7 (30%) |
| The navigation was consistent when moving between screens | 0 (0%) | 0 (0%) | 0 (0%) | 0 (0%) | 4 (17%) | 8 (35%) | 11 (48%) |
| The interface of the app allowed me to use all the functions such as entering information responding to reminders viewing information offered by the app | 0 (0%) | 0 (0%) | 0 (0%) | 2 (8.7%) | 1 (4.3%) | 9 (39%) | 11 (48%) |
| The app has all the functions and capabilities I expected it to have | 1 (4.3%) | 0 (0%) | 1 (4.3%) | 2 (8.7%) | 4 (17%) | 10 (43%) | 5 (22%) |
| **Usefulness (N = 23)** | | | | | | | |
| The app would be useful for my health and well being | 0 (0%) | 0 (0%) | 1 (4.3%) | 0 (0%) | 2 (8.7%) | 9 (39%) | 11 (48%) |
| The app helped me manage my health effectively | 1 (4.3%) | 0 (0%) | 1 (4.3%) | 1 (4.3%) | 5 (22%) | 6 (26%) | 9 (39%) |
| *^1^* n (%) | | | | | | | |
