# Supplementary material for: Evaluating User Experience and Satisfaction in a Concussion Rehabilitation App: Usability Study
Source: JMIR Form Res. 2025 Apr 11;9:e67275. doi: 10.2196/67275 (PMC12007725; doi:10.2196/67275)
Supplement: Multimedia Appendix 2 [file formative-v9-e67275-s002.docx]

*Multimedia Appendix 2. Summary of responses by participants with concussion to additional custom questions following a 2-week use period of a personalized concussion rehabilitation mobile application.*

| **Question (N = 23)** | **Strongly Disagree***^1^* | **Disagree***^1^* | **Somewhat Disagree***^1^* | **Neither Disagree nor Agree***^1^* | **Somewhat Agree***^1^* | **Agree***^1^* | **Strongly Agree***^1^* |
| --- | --- | --- | --- | --- | --- | --- | --- |
|  |  |  |  |  |  |  |  |
| I had confidence in the recommendations I received from the app | 0 (0%) | 0 (0%) | 1 (4.3%) | 4 (17%) | 2 (8.7%) | 9 (39%) | 7 (30%) |
| The flow of the exercise sessions on the app is easy to follow | 0 (0%) | 0 (0%) | 0 (0%) | 0 (0%) | 3 (13%) | 10 (43%) | 10 (43%) |
| I knew exactly which exercises I had to do during an exercise session | 0 (0%) | 0 (0%) | 0 (0%) | 0 (0%) | 4 (17%) | 4 (17%) | 15 (65%) |
| The in app voice commands are clear and understandable | 0 (0%) | 0 (0%) | 0 (0%) | 0 (0%) | 1 (4.3%) | 7 (30%) | 15 (65%) |
| The exercise report section is easy to follow and understand | 0 (0%) | 0 (0%) | 0 (0%) | 2 (8.7%) | 2 (8.7%) | 8 (35%) | 11 (48%) |
| *^1^* n (%) | | | | | | | |
